# Microbial mechanism of zinc fertilizer input on rice grain yield and zinc content of polished rice

**DOI:** 10.3389/fpls.2022.962246

**Published:** 2022-08-25

**Authors:** Yang Sean Xiao, Bo Zhou, Zhuangzhuang Han, Shenzhou Liu, Can Ding, Feifei Jia, Wenzhi Zeng

**Affiliations:** ^1^College of Water Resources and Civil Engineering, China Agricultural University, Beijing, China; ^2^Engineering Research Center for Agricultural Water-Saving and Water Resources, Ministry of Education, Beijing, China; ^3^State Key Laboratory of Water Resources and Hydropower Engineering Science, Wuhan University, Wuhan, China; ^4^Guangxi Hydraulic Research Institute, Nanning, China; ^5^College of Water & Architectural Engineering, Shihezi University, Shihezi, China

**Keywords:** rice, zinc application, bacterial community, co-occurrence network, microbial function

## Abstract

Zinc is an essential minor element for rice growth and human health, which can also change the structure of the microorganisms. However, it remains unclear for the effects of zinc fertilizer on microbiome function in agricultural soils and crops. To solve this research gap, we investigated the relationship between improving rice (*Oryza sativa* L.) yield, Zn concentration, soil microbial community diversity, and function by the application of Zn fertilizer. The field trials included three rice varieties (Huanghuazhan, Nanjing9108, and Nuodao-9925) and two soil Zn levels (0 and 30 kg ha^–1^) in Jiangsu province, China. As a test, we studied the variety of soil bacterial composition, diversity, and function using 16S rRNA gene sequencing. The results showed that soil Zn application reduced the diversity of microbial community, but the bacterial network was more closely linked, and the metabolic function of bacterial community was improved, which increased the grain yield (17.34–19.52%) and enriched the Zn content of polished rice (1.40–20.05%). Specifically, redundancy analysis (RDA) and Mantel’s test results revealed soil total nitrogen (TN) was the primary driver that led to a community shift in the rice rhizosphere bacterial community, and soil organic carbon (SOC) was considered to have a strong influence on dominant phyla. Furthermore, network analysis indicated the most critical bacterial taxa were identified as *Actinobacteria, Bacteroidetes, Proteobacteria*, and *Chloroflexi* based on their topological roles of microorganisms. KEGG metabolic pathway prediction demonstrated that soil Zn application significantly (*p* < 0.05) improved lipid metabolism, amino acid metabolism, carbohydrate metabolism, and xenobiotic biodegradation. Overall, their positive effects were different among rice varieties, of which Nanjing-9108 (NJ9108) performed better. This study opens new avenues to deeply understand the plant and soil–microbe interactions by the application of fertilizer and further navigates the development of Zn-rich rice cultivation strategies.

## Introduction

Rice (*Oryza sativa* L.), as the main source of zinc (Zn) intake for humans (Qin et al., 2012), is one of the most important food crops and staple food for more than one-third of the world’s population (Kasote et al., 2021) and is recognized as a major food crop in Asia due to its high demand and high planting rate (Nayar, 2014). However, Zn deficiency is a widespread micronutrient disorder in rice, resulting in reduced rice grain yield and poor nutrition quality (Fageria et al., 2002), continuous application of fertilizer and their low efficiency, especially N and P, and has caused environmental degradation (Bindraban et al., 2020). These problems have posed a major threat to sustainable rice production and food security (Rahman et al., 2022). Zn is one of the irreplaceable minor elements required for rice growth and other food crops (Broadley et al., 2007). It not only promotes plant metabolism, but also acts as an activator of cell division, protein synthesis, and gene expression (Yamaji et al., 2013). At the same time, as one of the essential micro-nutrients in the normal operation of various organs (Chasapis et al., 2012), Zn plays a vital role in human health (Ma et al., 2008). The average Zn content of rice in China is only 17 mg kg^–1^, which can be as low as 9.2 mg kg^–1^ in cooked rice (Chinese Center for Disease Control and Prevention). Considering the weight ratio, it was almost impossible to meet the recommended criteria for daily Zn intake (7.5–12.5 mg d^–1^) (Wang et al., 2016). The application of Zn fertilizer, such as zinc sulfate (ZnSO_4_), is a quite effective method to correct the deficiency of Zn and further improve in grain yield and increase grain Zn concentration (Khan et al., 2003; Rehman et al., 2018). Zn application methods mainly include soil application, foliar application, seed priming, and seed coating (Nadeem and Farooq, 2019; Nazir et al., 2021). Recent research has found that the application of 5–45 kg ZnSO_4⋅_7H_2_O ha^–1^ to the soil increased grain yield (13–60%) (Khan et al., 2002; Huang et al., 2019), while foliar application seems more effective in boosting grain Zn concentration (Fageria et al., 2009). In addition, other fertilizers (e.g., nitrogen fertilizer and bio-activated organic fertilizer), rice genotypes, and soil microorganisms are also influential factors in determining the Zn-rich capacity of rice (Ghasal et al., 2018; Wang et al., 2021). For instance, Hussain et al. (2020) used *Bacillus* sp. AZ6 as an inoculant of bio-activated organic fertilizers and found significant improvement in the crop growth, physiology, yield, and Zn content.

Soil microorganisms play important roles in plant performance by improving mineral nutrition, soil fertilizer, and soil quality (Weese et al., 2015; Gu et al., 2019). The nitrogen-fixing and nutrient mineralization processes carried by soil microorganisms can metabolize recalcitrant forms of N, P, and S to literate these elements for plants (Jacoby et al., 2017). In recent years, some scholars have proposed plant growth-promoting rhizobacteria (PGPR) as an alternative and eco-friendly technology that can enhance zinc solubilization and its availability to plants (Zeb et al., 2018; Bhatt and Maheshwari, 2020). Some bacteria, including *Acinetobacter, Bacillus*, and *Pseudomonas*, have been reported to solubilize zinc (Kumar et al., 2019). Thus, the production of biological fertilizers containing beneficial microorganisms may be an effective alternative to chemical fertilizers. In addition, potential interactions were found among bacterial communities, soil chemical properties, and crop growth status, which affected soil fertility and nutrient resorption characteristics of rice, leading to grain yield and Zn content variations in rice (Wang et al., 2021; Zhang et al., 2021). However, soil microorganisms are also shaped by many factors, such as farming manipulation, plant varieties, and soil nutrition (Berg and Smalla, 2009; Hartman et al., 2018). Changes of soil microbial community can be used to assess the quality of soil ecosystems (Pablo et al., 2014). Zn, as required nutrients for microorganisms, can be toxic to microorganisms by the displacement of essential metals from their native binding sites or through ligand interactions at high levels (Bruins et al., 2000). Co-occurrence network analysis can be used to explore changes in species interactions or microbial responses under environmental stress or human disturbance and to identify for key species (Deng et al., 2012; Bissett et al., 2013). However, soil microorganism and co-occurrence patterns to sustainable agricultural cultivation in rice are largely unknown.

In view of the above, we attempt to study the soil microorganisms, grain yield, and grain Zn concentration of rice affected by Zn fertilizer under sustainable agricultural practices (using microbial fertilizers instead of NPK fertilizer). The main objectives of this study are to test the following hypotheses: (i) the effect of Zn fertilizers differs among the common rice varieties in Jiangsu, China; (ii) the application of Zn fertilizers plays a significant role in shaping rhizosphere bacterial community and altering potential function. These findings obtained were beneficial to a more sustainable way of promoting Zn in rice through the regulation of the rhizosphere microbial community.

## Materials and methods

### Experimental background and zinc fertilizer input treatments

This study was conducted in Yancheng City, Jiangsu Province, China (33°11′ N, 119°52′ E). This area has a subtropical monsoon climate, the average annual air temperature is 15.45 ± 0.57°C, and the average annual precipitation is 1049.1 ± 304.1 mm (from 2011 to 2020; China National Knowledge Infrastructure, 2022). The value of available soil zinc in the field was detected as 1.07 ± 0.06 before the experiment. Three kinds of high-quality, high-yielding rice varieties (i.e., HHZ, Huanghuangzhan; NJ9108, Nanjing-9108; ND9925, Nuodao-9925) cultivated locally were chosen as the tested plants, and the critical limit of zinc (Zn) in the soil for rice ranges from 0.80 to 0.85 μg/g (Rahman et al., 2022). Two levels of basal Zn fertilizers (0 and 30 kg ha^–1^) were applied as ZnSO_4_⋅H_2_O ha^–1^ (effective Zn content 34.5%) before rice transplantation (25 May 2020). Therefore, six treatments were contained for this study. For each treatment, approximately 6000 m^2^ of field was divided into seven plots as replicates (see Supplementary Figure 1 for sampling diagram). Foliar Zn and microbial fertilizers were applied in all the treatments, where the foliar Zn application (3 kg ZnSO_4_⋅H_2_O ha^–1^ dissolved in 750 L of water) was sprayed at the booting stage, early grain-filling stage, and late grain-filling stage, respectively. The microbial fertilizers of 15,000 kg ha^–1^ [Manufacturer: Hebei Sanfeng Biofertilizer Co., Ltd; organic matter ≥ 70%, effective live microorganism (bacterial and fungal mix) ≥ 5 × 10^8^ g^–1^], which contained a high content of composite microorganisms, mainly composed of *Bacillus* spp. and *Trichoderma* spp. were applied with basal fertilizer before rice transplantation. All the experimental treatments had the same seed planting and transplanting times on 21 April 2020 and 30 May 2020, respectively. In each hole, 3–5 seedlings were transplanted. The planting density was converted to 30 holes per m^2^ uniformly, and irrigation and tillage management were consistent across all treatments. The details of treatments in this study are shown in Table 1.

**TABLE 1:** Treatments, abbreviations, and fertilization status.

Treatments	Cultivars^a^	Abbreviations	Basal application	Top-dressing	Live cycle
**Control group (ZS0)**	HHZ^b^	XRZS0	Microbial inoculum 15,000 kg ha^–1^		May 30–September 14
	NJ9108^c^	JRZS0			May 30–October 23
	ND9925 ^d^	NRZS0			May 30–October 23
				Apply 3 kg of ZnSO_4‘_H_2_O dissolved in 750 L of water per hectare at each application during the booting stage, early grain-filling stage, and late grain-filling stage, respectively	
**Zn-fertilizer treatments (ZS2)**	HHZ^b^	XRZS2	30 kg ZnSO_4‘_H_2_O ha^–1^ + Microbial inoculum 15,000 kg ha^–1^		May 30–September 14
	NJ9108^c^	JRZS2			May 30–October 23
	ND9925^d^	NRZS2			May 30–October 23

^a^HHZ, Huanghuazhan; NJ9108, Nanjing 9108; ND9925, Nuodao 99-25.

^b^HHZ is an indica rice that was harvested on 14 September 2020.

^cd^NJ9108 is a japonica rice and ND9925 is a glutinous rice, which both were harvested on 23 October 2020.

### Testing of rice yield and zinc content in polished rice

A 0.25 m^2^ sample square of consistently growing rice plants were randomly collected at maturity for each treatment per plot. All unfilled spikelets were removed, and others were recorded. The grain yield was de-enzyme using an oven at 105°C for 30 min and then dried at 70°C to a constant weight. Subsequently, the grains were rolled out, and the dry matter mass of the rice grain yield was weighed. Following this treatment, the grain yield was adjusted to the standard moisture content of 0.14g H_2_O g^–1^ fresh weight and converted to yield per hectare by area ratio.

We determined the zinc content in polished rice according to the current Chinese National Food Safety Standard—Determination of Zinc in Food (GB 5009.14-2017). Briefly, 2 g of polished rice was put in a conical bottle with 10 mL of nitric acid (HNO_3_) and 0.5 mL of perchloric acid (HClO_4_) and then carried out the digestion on an adjustable electric heating furnace. After digestion of the sample, the absorbance was measured at 213.9 nm by flame atomization, which then was quantitatively compared with the standard series.

### Rhizosphere sampling and chemical analysis

Samples of rhizosphere from each treatment group were collected from each replicate plot. For each plot, 5 sampling points were chosen in S-shaped pattern (Supplementary Figure 1), and a total of 10–12 rice plants from each sampling point were collected to ensure that the root system was as intact as possible. These five sub-samples were mixed well and used as one replicate. The sampled soil specimens included both loosely and tightly bound root soil. Loosely attached root soils from plots numbered 2, 4, and 6 were gently removed, passed through a 2 mm sieve, and then used to analyze the soil chemical properties (totally 18 samples). Tightly bound soils from plots 1–7 were separated using a sterile brush and then were re-suspended in 10 mL of a sterile 0.8% NaCl by vigorous shaking for 3 min (Angelo-Picard et al., 2004). These samples were used as rhizosphere soil (a total of 42 samples) and stored at –80°C for high-throughput sequencing. RDA and Mantel’s test were performed with bulk soils from plot numbered 2, 4, and 6 and rhizosphere soil from the same plots.

The soil chemical properties involved the soil pH, soil organic carbon (SOC), soil total nitrogen (TN), soil effective nitrogen (AN), soil total phosphorus (TP), soil total potassium (TK), cation exchange capacity (CEC), and soil available Zn (AZ) values. The Supplementary Part 1 provided the test methods, and the results are shown in Table 2.

**TABLE 2:** Zinc content in polished rice and grain yield of three rice cultivars.

Grain factor	Treatment^a^	Rice cultivar		
		HHZ	NJ91088	ND9925
Zinc content in polished rice (mg kg^−1^)	ZS0	20.03 ± 0.06 a^b^	27.93 ± 0.06 a	28.53 ± 0.08 a
	ZS2	21.37 ± 0.13 a	33.53 ± 0.03 b	28.93 ± 0.11 a
Grain yield (10^3^ kg ha^–1^)	ZS0	10.51 ± 0.14 a	9.56 ± 0.18 a	9.85 ± 0.13 a
	ZS2	12.72 ± 0.08 a	11.22 ± 0.08 a	11.78 ± 0.14 a

^a^ZS0, without zinc fertilization; ZS2, with basal zinc fertilization.

^b^Absolute number (mean value ± standard error) of zinc content in polished rice and grain yield analyzed by Duncan’s multiple comparisons. Different lowercase letters indicate that there was a significant difference between ZS0 to ZS2 (*p* < 0.05).

### Soil microbial and bioinformation analysis

#### DNA extraction, PCR amplification, high-throughput sequencing, and Real-time quantitative PCR

DNA was extracted from rhizosphere samples of six treatments (3 varieties × 2 Zn application levels; ca, 0.35 g), 42 samples in total (7 replicates per treatment), using a Fast DNA TM Spin Kit (MP Biomedicals LLC, United States) according to the manufacturer’s instructions. The V3-V4 region of the 16S rRNA gene fragment was amplified using a primer set 338F (5’ ACT CCT ACG GGA GGC AGC AG-3’) and 806R (5’-GGA CTA CHV GGG TWT CTA AT-3’) (Sangon Bioengineering, Co., Ltd, China). Paired-end format of purified amplicons was mixed well and sequenced using an Illumina Miseq sequencing platform according to the standard protocols by Shanghai Majorbio Bio-pharm Technology Co., Ltd (Shanghai, China). Flash (v 1.2.11^1^) was used to remove the primer sequences and contig paired ends. QIIME, an open-source bioinformatics pipeline (v 1.9.1^2^), was used to check the quality of the raw sequences. Clustering of high-quality sequences at 97% similarity to generate operational taxonomic units (OTUs) was conducted using UPARSE^3^. Valid sequences were matched against the SILVA database^4^ followed by a taxonomic comparison of representative OTU sequences at the 97% similarity level using the RDP classifier Bayesian algorithm (Stackebrandt and Goebel, 1994), with threshold of 70% was used to discriminate the phylum, order, family, and genus levels. A total of 9,500 OTUs were obtained by randomly sub sampling with the minimum number of sequences from the high-throughput test. Real-time quantitative PCR, targeting the bacterial 16S gene, was conducted with the primer sets Eub338 forward primer (5’-ACT CCT ACG GGA GGC AGC AG-3’) and Eub806 reverse primer (5’-GGA CTA CHV GGG TWT CTA AT-3’), pMD-18T (2692 bp, Takara Biotechnology Co., Ltd., Dalian, China) Easy vector was used to clone PCR products. The details are in Supplementary Part 2.

#### Co-occurrence networks construction

Co-occurrence network analysis of the rhizosphere bacterial communities under each treatment was conducted for six treatments (XRZS0, XRZS2, JRZS0, JRZS2, NRZS0, and NRZS2) with the same similarity threshold of 0.960 using the MENA platform on the Environmental Genomics Institute Website of the University of Oklahoma^5^ (Zhou et al., 2010). OTUs were screened and saved with the sequence numbers exceeding or equaling to 5 in at least three samples to filter for rare OTUs in each treatment. The results obtained from the MENA platform were visualized by Cytoscape (v 3.8.0) and Gephi software (v.0.9.2). The details can be found in the Supplementary Part 3.

#### Potential function prediction

The functional genes of rice rhizosphere bacteria were conducted by PICRUSt 2 (v 1.1.0^6^), and KEGG functional annotation was used to obtain information on the annotation of OTUs at each KEGG function level and abundance (Langille et al., 2013; Wu et al., 2022). STAMP software (v 2.1.3) was used for statistical hypothesis tests and exploratory plots (Parks et al., 2014) using Welch’s *t*-test to compare the differences in two groups.

### Statistical analysis

Soil chemical properties were recorded using Excel (v.2019). Two-way analysis of variance (ANOVA) was used to analyze whether basal Zn application, rice cultivars, and their interaction had a significant effect on soil chemical properties, grain yield, and Zn content in polished rice. The least significant difference (LSD) tests were performed at the *p* < 0.05 level. Alpha-diversity was calculated using Shannon, Evenness, and Sobs indices by MOTHUR (Schloss et al., 2009) (Wilcoxon rank-sum test. v 1.30.2^7^). The differences in the microbial community structure were analyzed using principal coordinate analysis (PCoA) based on the Bray–Curtis distance (Wang et al., 2019). The linear discriminant analysis effect size (LEfSe, Afgan et al., 2018) was determined to identify the key rhizosphere bacteria (Kruskal-Wallis test *p* < 0.05 and logarithmic LDA score (log10) > 3.7). Redundant and canonical correlation analyses were conducted to illustrate the relationships between rhizosphere bacterial communities and the soil chemical properties by Canoco 5 (Legendre et al., 2011). Spearman’s correlations between key microbial communities (genus level) and soil chemical properties aimed to determine the relationship between key genus and environmental variables (at *p* < 0.05 and *p* < 0.01, respectively).

## Results and analysis

### Shift in rhizosphere microbial community structure and biomarkers after zinc treatment

**FIGURE 1:**
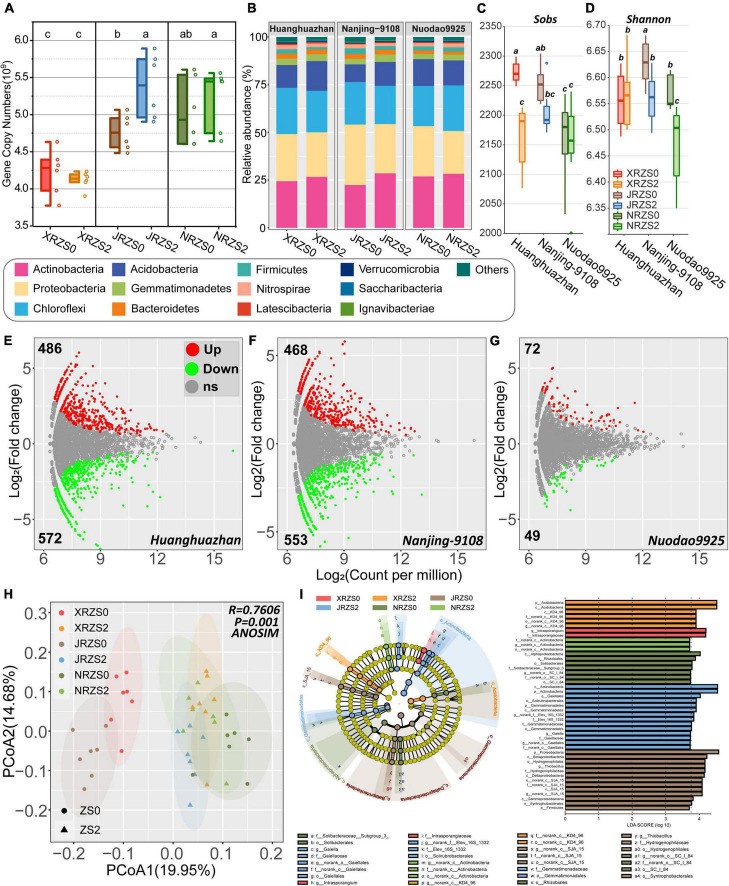
**(A)** Different soil Zn application and rice cultivars on the abundance of the rhizosphere soil bacterial gene copy numbers per gram sample; **(B)** microbial community composition of dominant bacterial phyla in rice rhizosphere soil. Phyla with a total relative abundance of < 0.5% are grouped in “Others”; **(C)** Sobs index; **(D)** Shannon index; **(E–G)** The volcano plot illustrates the up- and downregulated patterns of the rice rhizosphere bacterial microbiomes in different rice cultivars with Zn addition (ZS2), compared with control groups (ZS0). Each point represents an operational taxonomic unit (OTU), and the color represents up- and downregulated patterns of OTU. The *x*-axis represents the average abundance of OTU (as counts per million, CPM), and the *y*-axis represents the log_2_(fold change). **(E)** Huanghuazhan (HHZ), **(F)** Nanjing-9108 (NJ9108); **(G)** Nuodao9925 (ND9925). **(H)** Principal coordinate analysis (PCoA) of the rhizosphere soil bacterial community based on Bray–Curtis distance at the OTU level. The ellipses represent the 95% confidence intervals of each treatment; **(I)** bacterial biomarkers in different treatments and cultivars based on linear discriminant analysis (LDA) using LEfSe analysis. Different colors represent different treatments, and the circles from inside to outside correspond with phyla to genus. Colors coded within the cladogram indicate the taxa with different abundances in treatment by the Kruskal–Wallis test with *p* < 0.05 and logarithmic LDA score (log10) > 3.7. Genera with a relative abundance of less than 0.2% are not included.

As shown in Figure 1A, 16S rRNA gene abundance (per g of soil) for XRZS2 treatment (5.223 × 10^9^) was 2.56% lower than XRZS0 (5.383 × 10^9^), while JRZS2 and NRZS2 were both higher than the control treatment group (5.383 × 10^9^ vs. 4.762 × 10^9^ and 5.223 × 10^9^ vs. 5.037 × 10^9^), resulting in JRZS2 increasing by 13% and significantly differing from JRZS0 (*p* < 0.05, ANOVA Duncan’s test).

A total of 2,412,863 (57,449 ± 7487/sample) microbial 16S V3-V4 valid reads were obtained from 42 samples through high-throughput sequencing analysis and classified into 9,500 operational taxonomic units (OTUs) at the 97% similarity level. The rarefaction curve indicated that the sequence depths of all tested samples were adequate for further analysis (Supplementary Figure 1). A total of 38 different bacterial phyla were found in the different treatment groups. Taxonomic analysis revealed that the rhizosphere of rice mainly included five phyla (Figure 1B), among which *Actinobacteria* (22.45–28.54%) was the most dominant phylum followed by *Proteobacteria* (22.44–31.67%), *Chloroflexi* (20.17–24.25%), and *Acidobacteria* (9.26–15.53%). After Zn treatment, *Actinobacteria* and *Gemmatimonadetes* significantly increased (5.22–27.09% and 6.36–33.40%, respectively), while *Proteobacteria* and *Firmicutes* decreased by 5.49–18.50% and 4.67–46.44%, respectively (*p* < 0.05, Wilcoxon rank-sum test).

The richness, diversity, and evenness of the bacterial community exhibited a similar decrease trend after soil Zn fertilizer application (ZS2). The sobs index of rhizosphere microorganisms of the three kinds of rice decreased by 0.4–4.92%, with HHZ and NJ9108 showing a significant difference (*p* < 0.05, Wilcoxon rank-sum test) (Figure 1C). While the Shannon diversity index decreased by 0.11–1.60%, only HHZ did not exhibit a discernible change (Figure 1D). The PCoA based on the Bray–Curtis distance showed that the structure of the bacterial communities differed significantly among the two kinds of soil Zn application treatments (Expect for ND9925) and the three tested cultivars (*p* = 0.001). Principal components explained 34.63% of all the differences in the data (Figure 1H).

The volcano plot illustrates the variance of bacterial abundance after Zn treatment (Figures 1E–G), large variation in bacterial community was observed in HHZ and NJ9108 with 11.70% (1058/9500) and 10.81% (1027/9500) OTUs changing significantly, while only 1.3% (121/9500) OTUs were observed in ND9925 treatment, which confirms the previous results. Differential OTUs mainly from *Anaerolineaceae* and *Acidobacteria* (family) were both significantly enriched and depleted in three treatments. In addition, the family *Gemmatimonadaceae* exhibits significant enriched and overlapped in three treatments.

To explore the importance of key species more accurately, LEfSe analysis was conducted to identify the biomarkers of bacterial communities in different treatments at the class level (Wilcoxon rank-sum, *p* < 0.05, LDA score > 3.7). A total of 45 bacterial classifications were enriched in the six treatments. At the class level, XRZS0 was notably enriched in *Acidobacteria KD4_96* after applying Zn fertilizer (XRZS2). JRZS0 was significantly enriched in *Gammaproteobacteria, Deltaproteobacteria*, and *Betaproteobacteria* after applying soil Zn fertilizer (JRZS2). It was also significantly enriched in *Actinobacteria* and *Gemmatimonadetes* at the class level. NRZS0 was enriched in *Alphaproteobacteria*, while NRZS2 was not enriched. Zn fertilizer treatment enriched the phyla of *Acidobacteria, Actinobacteria*, and *Gemmatimonadetes*, families of *Gaiellaies* and *Gemmatimonadaceae*, and the genera of *Gaiella* (Figure 1I).

### Dynamic changes of co-occurrence network association

**FIGURE 2:**
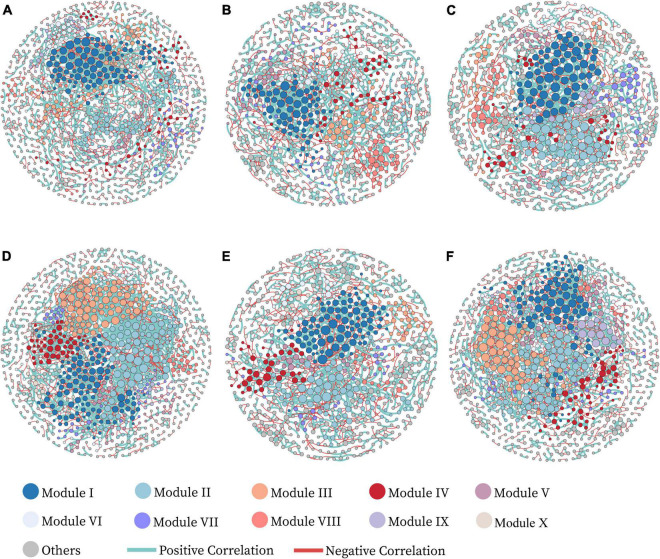
Visualized networks of microbial co-occurrence in different treatments, **(A–F)** represent the networks of treatments XRZS0, JRZS0, NRZS0, XRZS2, JRZS2, and NRZS2, respectively. The nodes are colored by module. The module eigengenes from the top 10 large sub-modules explained 41.25–58.81% of the variations of the relative abundance across different replicates, suggesting that these eigengenes represented the module profiles relatively well. Nodes represent an operational taxonomic unit (97% sequence identifies a threshold, OTU). The size of each node is proportional to the node degree. The edges in green and red represent co-occurrence and mutual exclusion patterns among taxa.

We established six co-occurrence networks to further characterize the influence of Zn application and tested cultivars on bacterial networks (Figure 2 and Table 3). In the treatments without soil Zn application (ZS0), OTUs were mainly clustered into the top two modules, while the number of modules gradually increased with soil Zn application. For HHZ and NJ9108, the number of nodes in the group treated with soil Zn fertilizer decreased slightly (915 vs. 898 and 960 vs. 906), while the number of nodes in ND9925 remained similar (863 vs. 864). Compared to the controls, the number of total links was significantly higher (1546 vs. 1987 and 1382 vs. 1680) and the average path length was lower (11.905 vs. 8.656 and 11.263 vs. 9.485) in the HHZ and ND9925, amounting to no significant difference in any of the NJ9108 treatments. In addition, the higher avgK and avgCC were observed in ZS2. In summary, soil Zn fertilizer application had little effect on HHZ, but increased the complexity of all the bacterial networks.

**TABLE 3:** Topological features of the co-occurrence network of soil microbial communities of the rhizosphere of rice with different treatments and cultivars.

Treatments	Abbreviations	Empirical network								Random network^a^		
		Similarity threshold (St)	Network size(n)	Total links	*R*^2^ of power-law	Average Connectivity (avgK)	Average clustering coefficient (avgCC)	Average path distance (avgGD)^b^	Modularity (M)	Average clustering coefficient (avgCC)	Average path distance avg (GD)	Modularity (M)
**Control groups** **(ZS0)**	XRZS0	0.960	915	1546	0.880	3.379	0.339	11.905	0.855	0.006 ± 0.002	5.182 ± 0.043	0.589 ± 0.004
	JRZS0	0.960	960	1474	0.829	3.071	0.348	10.950	0.911	0.004 ± 0.002	5.748 ± 0.047	0.636 ± 0.004
	NRZS0	0.960	863	1382	0.840	3.203	0.347	11.263	0.854	0.005 ± 0.002	5.440 ± 0.046	0.613 ± 0.005
**Zn-fertilizer treatments** **(ZS2)**	XRZS2	0.96	898	1987	0.798	4.425	0.369	8.656	0.781	0.009 ± 0.002	4.377 ± 0.028	0.476 ± 0.004
	JRZS2	0.960	906	1432	0.809	3.161	0.358	10.965	0.883	0.004 ± 0.002	5.590 ± 0.048	0.622 ± 0.004
	NRZS2	0.960	864	1680	0.830	3.889	0.353	9.485	0.872	0.007 ± 0.002	4.795 ± 0.033	0.529 ± 0.004

^a^Random networks were generated by rewiring all nodes and links corresponding to empirical networks 100 times.

^b^GD, geodesic distance.

**FIGURE 3:**
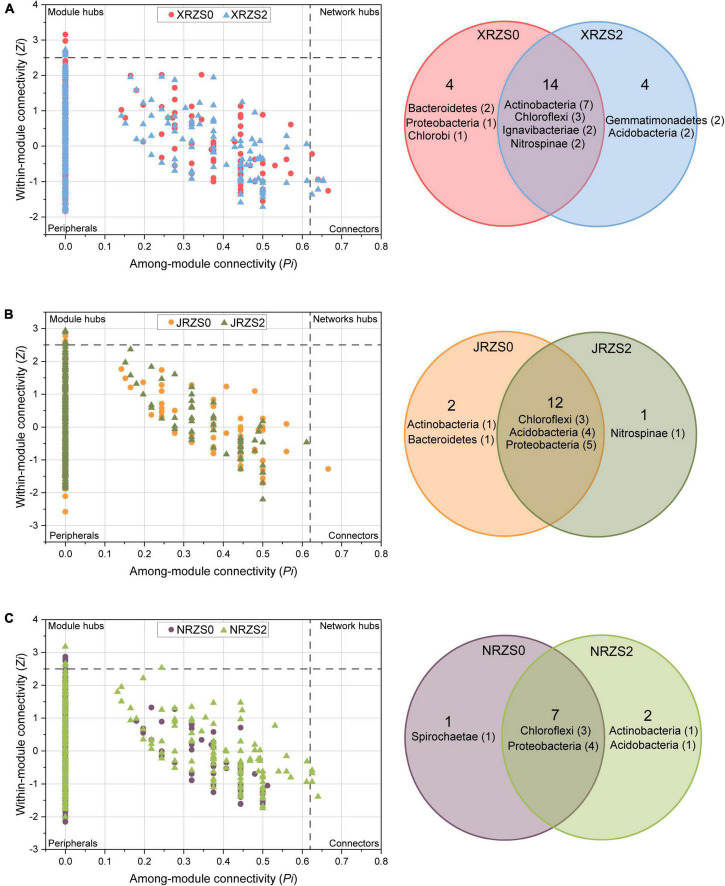
*Z*_*i*_-*P*_*i*_ plot showing the distribution of OTUs based on their topological roles representing an OTU with (ZS2, triangle) or without (ZS0, circle) basal zinc fertilizer. The threshold values of *Z*_*i*_ and *P*_*i*_ for categorizing OTUs are 2.5 and 0.62. The Venn diagram shows the similarities and differences between keystone hubs in networks between two treatments. **(A)** HHZ; **(B)** NJ9108; and **(C)** ND9925.

The topological roles of the OTUs are shown in Figure 3. The results demonstrated that most of the nodes (99.11%) were in the peripheral region, and their connections were mainly connected to the nodes in the module. All the nodes with *Z_*i*_* ≥ 2.5 or *P_*i*_* ≥ 0.62 were determined as the keystone species. Therefore, nodes in the area of connectors (0.24%) and module hubs (0.65%) played a crucial role in the co-occurrence networks. See Supplementary Table 1 for details concerning the bacteria classified as nodes in the connector and module hub area. Among them, the connectors and module hubs of phyla *Actinobacteria* (e.g., *g__Mycobacterium, g__Iamia*, and *g__Nocardioides*), *Proteobacteria* (e.g., *g__Nitrosospira, g__Steroidobacter, g__Shinella*, and *g__Geobacter*), and *Chloroflexum* (e.g., g*__Anaerolinea*) were accounted for 15.2, 16.9, and 15.3%, respectively. Thus, these were the most important species in the rhizosphere. Furthermore, in the group ZS2, the number of *Bacteroidetes* (e.g., *g__Chitinophaga* and *g__Pontibacter*) in the connectors and module hubs decreased from 5% in the ZS0 to 0, whereby applying Zn fertilizer reduced the relative abundance of *Bacteroidetes*, considered as key bacteria, indicating that the species belonging to the phylum *Bacteroidetes* were more sensitive to Zn fertilizer. Intriguingly, as shown in Figure 3, *Bacteroidetes* were all derived from HHZ and NJ9108. Applying soil Zn fertilizer significantly changed the topological structure of the key bacteria communities in the rice rhizosphere.

### Potential functional prediction of bacterial community

**FIGURE 4:**
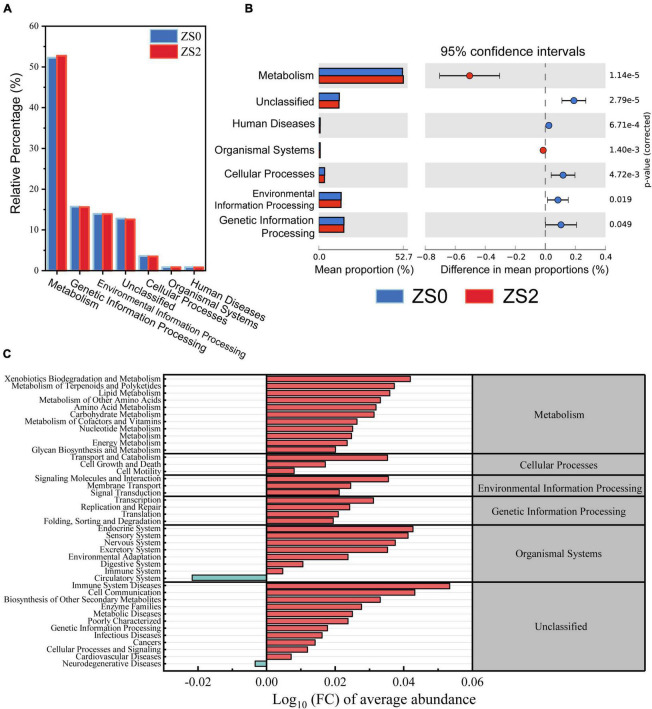
**(A)** Relative percentage of predicted functional gene abundance (at L1 level by KEGG); **(B)** KEGG categories (L1 level) differing significantly between ZS0 and ZS2 based on Welch’s *t*-test, the mean proportion of majority pathway are overabundant within the rhizosphere soil of ZS0 compared to ZS2; **(C)** expression abundance multiples of bacterial function at the KEGG L2 level, most functions of ZS2 were upregulated compared to ZS0.

The effect of basal zinc fertilizer application on the metabolic function of soil bacterial communities was investigated. The relative abundance of KEGG pathway (L1 level) in all samples is shown in Figure 4A. The main classifications include (ZS0 vs. ZS2) metabolism (52.24% vs. 52.74%), genetic information processing (15.70% vs. 15.60%), environment information processing (13.96% vs. 13.88%), unclassified (12.78% vs. 12.6%), cellular processes (3.61% vs. 3.5%), organismal systems (0.84% vs. 0.86%), and human diseases (0.85% vs. 0.82%). The results showed that all the above functions produced significant differences between ZS0 and ZS2, with mean proportion of metabolism and organismal systems being upregulated and others downregulated (Figure 4B).

To investigate in more detail the functional changes of zinc-rich processes, we calculated expression abundance multiples based on L2 level (Figure 4C). Overall, almost all functions were upregulated in all classifications, for example, lipid metabolism, amino acid metabolism, carbohydrate metabolism, xenobiotic biodegradation and metabolism functions, and membrane transport, while only two functions were downregulated in the circulatory system of organismal systems and neurodegenerative diseases of unclassified, respectively. The results above indicated that the application of exogenous Zn fertilizer altered the potential functions of microorganisms, which may positively affect bacterial functions.

### Variations in polished rice zinc content, grain yield, and soil chemical properties

The Zn content of polished rice and grain yield is shown in Table 2. Compared with the corresponding ZS0, the Zn content of polished rice in the XRZS2, JRZS2, and NRZS2 soil Zn application groups increased by 6.66, 20.05, and 1.40%, respectively, which was consistent with the variation trend of the soil available zinc content (*p* < 0.05). NJ9108 was the only one that differed significantly (*p* < 0.05). ZS2 had a relatively higher overall increase in yield. The yield of varieties HHZ, NJ9108, and ND9925 increased by 21.00, 17.34, and 19.52% after Zn treatment, respectively. Two-way ANOVA (Supplementary Table 1) suggested that the Zn content in polished rice and grain yield was significantly affected by cultivar differences and Zn application differences, respectively.

**FIGURE 5:**
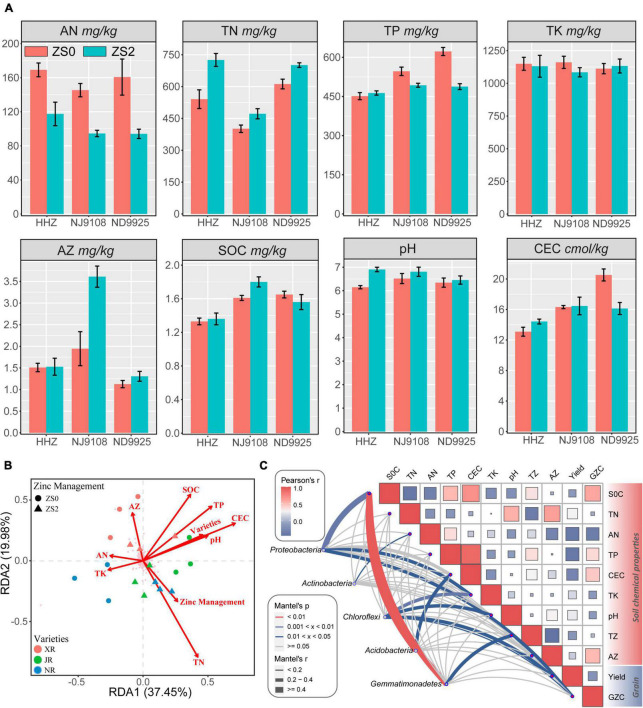
**(A)** Soil properties at the top 15 cm layer in the experimental fields. SOC, soil organic carbon; TN, total nitrogen; AN, available nitrogen; TP, total phosphorus; TK, total potassium; CEC, cation exchange capacity; AZ, available zinc; ZS0, without basal zinc fertilization; ZS2, with basal zinc fertilization. **(B)** The relation among the bacterial community, environmental parameters, and varieties of rice and soil Zn application. Redundancy analysis (RDA) of the correlation between the bacterial community (OTU level) and soil chemical properties. Red lines represent significant factors, and the pink crosses represent OTUs; **(C)** correlations between soil chemical properties, grain yield, grain zinc content (GZC), and dominant bacterial phyla (with a total relative abundance > 3%). The relative abundance of dominant bacterial phyla based on Bray–Curtis distance is related to other factors by Mantel’s test. Line width indicates the Mantel’s r statistic, and color denotes the significance based on 999 permutations. The color gradient in heatmap corresponds to Pearson’s correlation coefficient.

The shift in soil chemical properties among different treatments is provided in Table 4. The soil Zn application increased the soil total nitrogen (TN) by 14.58–34.20% and pH by 1.74–12.20%, but it dramatically decreased the available nitrogen (AN) by 30.56–41.48% and total phosphorus (TP) content (Figure 5A). Cultivar, treatment, and their interactions influenced almost all soil chemical characteristics. However, no effect was observed on total potassium (TK) concentration. Compared with the effect of cultivar, treat affected soil pH significantly, while cultivar had a significant effect on SOC. In addition, the interaction term cultivar × treat had no effect on AN.

**TABLE 4:** Two-way ANOVA for soil properties as affected by basal Zn application (treat), rice cultivar (cultivar), and the interaction (treat × cultivar).

Soil properties^a^	Cultivar		Treat		Interaction	
	*P*-value	*F*-value	*P*-value	*F*-value	*P*-value	*F*-value
**SOC**	<0.001***	40.199	0.223	1.649	0.014*	6.185
**TN**	<0.001***	79.789	<0.001***	54.160	0.024*	5.152
**AN**	0.041*	4.231	<0.001***	70.789	0.572	0.586
**TP**	<0.001***	63.277	<0.001***	66.627	<0.001***	34.92
**CEC**	<0.001***	41.210	0.036*	5.566	<0.001***	17.919
**TK**	0.870	0.141	0.439	0.640	0.465	0.816
**pH**	0.124	2.492	0.002**	16.016	0.047*	4.001
**AZ**	<0.001***	58.250	<0.001***	24.593	<0.001***	17.536

^a^SOC, soil organic carbon; TN, total nitrogen; AN, available nitrogen; TP, total phosphorus; TK, total potassium; CEC, cation exchange capacity; AZ, available zinc; ZS0, without basal zinc fertilization; ZS2, with basal zinc fertilization.

^b^* Indicates a significant difference at the *p* < 0.05 level, ** indicates a significant difference at the *p* < 0.01 level, and *** indicates a very significant difference at the *p* < 0.001 level based on two-way ANOVA, LSD test. All significant differences are shown with bold.

### The impact of environmental factors on bacterial community

Redundancy analysis (RDA) was conducted to investigate the drivers of change occurring in the microbial community structures (Figure 5B). All the edaphic variables explained up to 57.43% of the variance, with the first axis explaining 37.45% and the second axis explaining another 19.98%. The RDA plots indicate that TN (27.6%, *F* = 6.1, *p* = 0.002) resulted in the longest vector followed by SOC, TP, and AZ. Meanwhile, TN had a positive influence on the presence of *Acidobacteria* and *Gemmatimonadetes* during the Zn treatment process. In addition, Mantel’s analysis was conducted between dominant phyla, environmental factors, grain yield, and zinc content in polished rice (Figure 5C). The results showed that the phyla *Proteobacteria, Acidobacteria*, and *Gemmatimonadetes* were significantly affected by SOC; among them, *Proteobacteria* and *Chloroflexi* were also affected by TK and pH as well, which may have potential impact on grain yield. According to Mantel’s *p* and *r* statistics, soil SOC affects dominant phyla more than other environmental factors. Overall, TN appeared to be the critical chemical factor for assembling the microbial community structure of the rice rhizosphere, while SOC seemed to have strong influence on dominant phyla.

## Discussion

### Zn fertilizer applications changed soil properties and bacterial community characteristics

As we all know, the availability of Zn is significantly influenced by soil pH (Zeng et al., 2019), and Zn deficiency is more common in high pH soil (Welch and Graham, 2005). However, in this study, we found higher pH and lower TP after soil Zn application. Xu et al. (2003) reported that submerged conditions increase the pH of acidic soil and decrease the pH of alkaline soil. In addition, Hacisalihoglu (2020) reported the higher solubility of P than Zn, resulting in a shift of Zn from soluble to insoluble Zn (e.g., ZnS). These reports could be the reason for this result, which may also lead to a slight increase in soil available Zn and limit the uptake of zinc in rice. In view of Chen Y. L. et al.’s (2022) report, the available Zn accumulated with the number of years of ZnSO_4_ application, and the period of this study was also a limiting factor for effective Zn. Prasad et al. (2010) reported that higher Zn increases biomass production, resulting in the addition of large quantities of roots and stubbles, which perhaps build up SOC content. Our result is in line with Prasad. Soil microorganisms served as the major drivers in SOM and nutrient cycling (Rath and Rousk, 2015), whose composition and activity characteristics significantly influence soil nutrient transfer and accumulation (Zheng et al., 2018; Luo et al., 2019). The higher 16S gene copy number might in part explain why SOC content is higher in soil. In addition, SOM has a high CEC (Kleber et al., 2015) which can explain the CEC change trend as SOM. Intriguingly, the content of total nitrogen was opposite to the change of available nitrogen. This may be due to the fact that Zn fertilization reduces the ability of the soil to convert organically bound N to inorganic form.

Furthermore, plant grain nutrient status and grain yield are significantly impacted by nitrogen, including the transportation of Zn in plants (Xue et al., 2019). Gupta et al. (2016) identified the increased root zone translocators and other organic compounds as major drivers that can accelerate the transport and accumulation of Zn in the xylem through N-stimulated activities. The internal relationship between nitrogen and Zn has a remarkable synergistic effect in regulating the Zn concentration in rice grains. This also corresponded to the results that TN was the most critical soil factor during the process. The results of this study demonstrated a lower increase in yield and Zn content for polished rice than the results of previous studies (Khan et al., 2003; Phattarakul et al., 2012; Khampuang et al., 2021). This could be owing to the fact that no NPK fertilizer was used in this study, which has a positive effect on Zn enrichment (Xue et al., 2019).

Previous studies found applying Zn fertilizer did not significantly change the alpha diversity of bacterial community (Garcia-Gomez et al., 2018). However, the Shannon index of HHZ was observed not to change significantly, while that of NJ9108 and ND9925 decreased significantly. It is speculated that excess Zn suppresses or kills the Zn-sensitive species within a short time (e.g., *Bacteroidetes*), resulting in a decrease of the Sobs index (Zeng et al., 2011). As the accumulated time of Zn fertilizer increases, more microorganisms will become dominant species, thereby lowering the evenness index. After that, competition among the dominant microorganisms may further accelerate the extinction of some bacteria (Zhang et al., 2021), thus decreasing the richness index. The average size of networks (n) decreased after Zn application, in accordance with diversity, but the average links and average connectivity (avgK) increased. This was because microorganisms often form complex networks and change their responses to external disturbances (Jiao et al., 2019; Wu et al., 2022). In addition, species that are highly interconnected and interact frequently are grouped into modules. The modularity of ZS0 is higher than ZS2, one explanation is that Zn application may reduce the significance of the rhizosphere difference in rice (Ling et al., 2022).

### Microbial mechanism of Zn fertilizer treatments and rice varieties promoted rice yield and zinc content

Chen Y. L. et al. (2022) reported that *Actinobacteria* thrived but *Bacteroidetes* were suppressed after 3 years of Zn application. Our results are exactly in line with it. A high percent abundance of Actinobacteria was observed in ZS2 treatments, which is associated with dissolving P and Zn in the soil, increasing the utilization ratio of P and Zn by plants (Rose et al., 2013). The families of *Gaiellaies* and the genera of *Gaiella* belonging to *Actinobacteria* are significantly enriched in this study, which are considered to have high Zn adaptability and storage potential (Pasti et al., 1990). In contrast, *Bacteroidetes* as a keystone species, selected by its topological roles, demonstrated suppression in ZS2 treatments, suggesting it is sensitive to Zn application. Based on the keystone species (genus level) selected, we used a correlation heat map based on Pearson’s coefficient to explore the interactions of soil chemical parameters and key genera (Supplementary Figure 5). The results indicated that the abundance of a single keystone had less correlation with the soil chemical parameters and grain yields, while Wang et al. (2021) also obtained similar results; it is speculated that these members of the bacterial community work together to affect the entire soil–plant system rather than acting independently.

**FIGURE 6:**
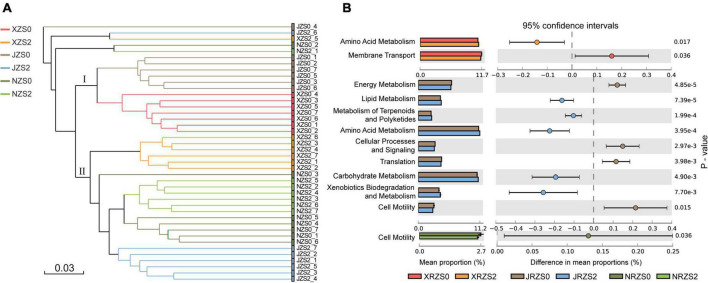
**(A)** Clustering analysis of bacterial communities under three rice cultivars and two soil Zn applications based on OTU abundance. Bray–Curtis similarity coefficients were used; **(B)** KEGG metabolism pathway (L2 level) differing significantly between ZS0 and ZS2 among three different rice cultivars (HHZ, NJ9108, and ND9925) with an effect size ≥ 10%.

The cluster analysis was conducted to explore the differences between rice varieties (Figure 6A). The results demonstrated that all treatments could be roughly divided into two groups. The first group contained XRZS0 and JRZS0 without soil Zn application, and the second group contained three Zn application treatments (ZS2) but NRZS0 without soil Zn application also merged. Overall, the structure of the bacterial community in the rice rhizosphere largely depended on whether soil Zn fertilizer was applied, followed by rice genotypes, which may have further influenced the grain yield and Zn concentration. Wissuwa et al. (2008) showed that rice yield and Zn content in polished rice varied with rice cultivars under the same growth conditions and were closely related to the different Zn absorption and utilization efficiency (White and Broadley, 2005; Song et al., 2015).

Function predictions among the three tested cultivars (Figure 6B) showed that the metabolism functions of NJ9108 improved better, with significant improvement in lipid metabolism, amino acid metabolism, carbohydrate metabolism, and xenobiotic biodegradation metabolism. Lipid metabolism, as a resistance of microbial cells to environment, may be affected by alterations in gene expression during stress acclimation phase (Kosová et al., 2018). Amino acid metabolism is a primary metabolic process driving microbial metabolism and biosynthesis, which facilitates microbial utilization of amino acid. Amino acids are the main form of organic nitrogen, and its degradation is beneficial for microbial reproduction (Dai et al., 2015; Idrees et al., 2020). Carbohydrate metabolism is related to the biosynthesis of sugars and the decomposition of organic matter, which may be influenced by the different cellulose, xylan, and fructan in plant material of different rice species (Berlemont and Martiny, 2015). Furthermore, membrane transport and cell motility pathway may be related to the input or output of zinc ions and ensure the efficient accumulation and distribution of zinc ions in the cell (Navarrete et al., 2017). These variations in rice cultivars are in good agreement with Chen W. et al. (2022). The downregulated expression in HHZ and ND9925 treatments leads to low nutrient absorption (Kisand et al., 2012), consequently leading to low Zn absorption.

### Limitations of the study

The findings of this study have to be seen in light of some limitations as follows:

(1) In order to reduce the application of chemical fertilizer, microbial fertilizers were applied as basal fertilizer. Inoculation of microorganisms into the soil may interfere with the interaction between native microorganisms and exogenous Zn fertilizers. Therefore, the shifts of microbial assemble and interactions among species are not exclusively a function of zinc application.
(2) Due to the outbreak of COVID-19 and time constraints, we only conducted a 1-year field trial. This study offered only a glimpse of the variation of soil microorganism and rice growth with soil Zn application. However, the environment, plants, and soil microorganisms all have variability and plasticity in response to the application of Zn or microbial fertilizer. It is necessary to conduct a long-term field trial to test the results.
(3) Based on the farm records of previous studies those conducted on these fields (blocks) involved in this study, there were no significant differences. However, as we did not apply randomized block design, the effects due to variations among different fields (blocks) could not be completely eliminated. Randomized block design would be a better choice in future studies, to minimize the effects of unpredictable variables on the results.

## Conclusion

This study focuses on the effects of soil Zn fertilizers and rice varieties on the grain yield, grain Zn concentrations, bacterial community, and co-occurrence patterns under sustainable farming manipulation. We found that the Zn treatments (ZS2) decreased the soil bacterial diversity, strengthened the network interactions, and enhanced the yield and zinc concentration in grain. Soil total nitrogen (TN) and soil organic carbon (SOC) were identified as the primary drivers that led to shifts in community and dominant phyla, respectively. In addition, the potential functions of microorganisms were improved in ZS2 treatment. This is a novel insight to reveal the bacterial assemble and co-occurrence patterns under sustainable farming manipulation with different soil zinc application levels. Further studies should be conducted to gain a better understanding of key microbial communities and Zn enrichment mechanisms in rice by combining metagenomic and transcriptomic data and finally explore ways to guide these microorganisms in rice farming, which may contribute to Zn-rich rice cultivation strategies and achieve sustainable agriculture.

## Data availability statement

The datasets presented in this study can be found in online repositories. The names of the repository/repositories and accession number(s) can be found below: NCBI – PRJNA845976.

## Author contributions

YX: data analysis and writing original draft—revising and editing. BZ: conceptualization, resources, reviewing and editing, supervision, and funding acquisition. ZH, CD, and FJ: data analysis and curation, and reviewing and editing. SL: field experiment, and reviewing and editing. WZ: resources, and reviewing and editing. All authors contributed to the study conception and design, commented on previous versions of the manuscript and approved the final manuscript.

## Footnotes

1
https://ccb.jhu.edu/software/FLASH/

2
http://qiime.org/install/index.html

3
http://www.drive5.com/uparse

4
https://www.arb-silva.de/

5
http://ieg2.ou.edu/MENA

6
http://picrust.github.io/picrust/

7
https://www.mothur.org/wiki/Download_mothur

## Abbreviations

TN: soil total nitrogen
AN: soil available nitrogen
TP: soil total phosphorus
TK: soil total potassium
CEC: soil cation exchange capacity
SOC: soil organic carbon
AZ: soil available zinc
PCoA: principal coordinate analysis
RDA: redundancy analysis
LEfSe: linear discriminant analysis effect size
LDA: linear discriminant analysis
OTU: operational taxonomic unit
HHZ, NJ908 and ND9925: Huanghuazhan (Japonica rice), Nanjing-9108 (Japonica rice), and Nuodao99-25 (glutinous rice)
ZS0 and ZS2: treatments without added basal zinc fertilizer and with basal zinc fertilizer.
