# Supplementary material for: Microbial mechanism of zinc fertilizer input on rice grain yield and zinc content of polished rice
Source: Front Plant Sci. 2022 Aug 25;13:962246. doi: 10.3389/fpls.2022.962246 (PMC9458200; doi:10.3389/fpls.2022.962246)
Supplement: Supplementary file 1 [file Data_Sheet_1.docx]

Supplementary Material

**Part 1 Test methods of soil chemical properties**

Soil chemical parameters and methods of determination were conducted as follows: the pH was measured using a potentiometric method for soil-water mixtures (soil:water=1:2.5, w/w). The soil organic carbon (SOC) content was determined by oxidation-spectrophotometry with potassium dichromate (TU-1901, China). The soil total nitrogen (TN) content was determined using the Kjeldahl method (automatic nitrogen tester KDN-103A, China). The total phosphorus (TP) content was determined by the perchloric acid and sulphuric acid-molybdenum antimony anti-colorimetric method (Visible Spectrophotometer Type 721, China). The total potassium (TK) content was determined by hydrofluoric acid digestion (AA-7003 series fully automatic flame/graphite furnace atomic absorption spectrophotometer, China). The cation exchange capacity (CEC) was determined using the ammonium chloride-ammonium acetate exchange method. The soil available Zn (AZ) was elucidated by the dithizone colorimetric method, according to the Chinese National Standard HJ 962-2018, HJ 615-2011, HJ 717-2014, NY/T 88-1988, GB 9836-88, LY/T 1243-1999, and LY/T 1261-1999, respectively. The effective soil nitrogen (AN) was determined by the alkaline nitrogen solution method. Soil properties were quantified by Pony Testing International Group (Beijing, China).

**Supplementary table 1 |** Two-way ANOVA for plant index (grain yield and Zn content in polished rice) as affected by basal Zn application (treat), rice cultivar (cultivar), and the interaction (treat × cultivar)

| **Plant index** | **Factor** | ***F* value** | ***p* value** |
| --- | --- | --- | --- |
| **Grain yield** | Treat | 6.016 | 0.030* |
|  | Cultivar | 0.832 | 0.459 |
|  | Interaction | 0.041 | 0.960 |
| **Zn content in polished rice** | Treat | 3.701 | 0.078 |
|  | Cultivar | 23.290 | <0.001*** |
|  | Interaction | 1.587 | 0.245 |

* indicates a significant difference at the *p*<0.05 level, *** indicates a very significant difference at the *p*<0.001 level based on two-way ANOVA, LSD test.


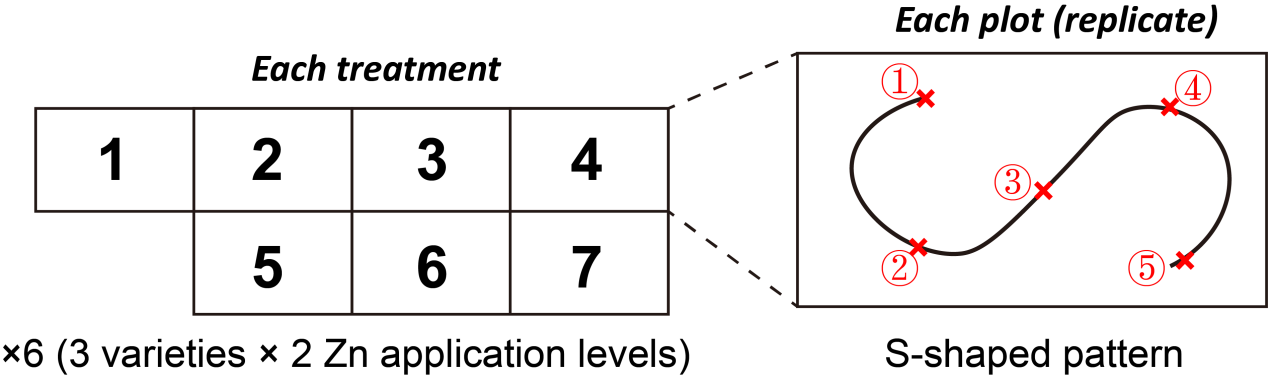


**Supplementary Figure 1 |** Sampling diagram

**Part 2 16S rRNA sequencing**

1. **DNA extraction and PCR amplification**

DNA was extracted from the six treatments rhizosphere sample (ca, 0.35 g), 42 samples in total (7 replicates per treatment), using a Fast DNA TM Spin Kit (MP Biomedicals LLC, USA) according to the manufacturer’s instructions. The V3-V4 region of 16S rRNA gene fragment were amplified using a primer set 338F (5’ ACT CCT ACG GGA GGC AGC AG-3’) and 806R (5’-GGA CTA CHV GGG TWT CTA AT-3’) by an ABI GeneAmp® 9700 PCR thermocycler (ABI, CA, USA, Sangon Bioengineering, Co., Ltd, China). Paired-end format of purified amplicons were mixed well and sequenced using an Illumina Miseq sequencing platform according to the standard protocols by Shanghai Majorbio Bio-pharm Technology Co., Ltd (Shanghai, China). The DNA concentration and quality were evaluated by a NanoDrop Spectrophotometer (Thermo NanoDrop 2000, Thermo Fisher Scientific Pte Ltd, USA) using OD260/230 and OD260/280. All DNA extracts results were assessed and stored at -80℃ for further amplification (Zhang et al., 2017). The PCR conditions were as follows: 3 min at 95℃, followed by 27 annealing cycles of 30 s at 95℃, 30 s at 55℃, 45 s at 72℃ and 10 min at 72℃, 10℃ until halted by the user. The PCR amplification used TransGen AP221-02: TransStart Fast pfu DNA Polymerase in a 20-μL volume system, which included 4.0 μL of 5× Fast Pfu Buffer, 2.0 μL of 2.5 μM dNTPs, 0.8 μL of each primer (5.0 μM), 0.4 μL of Fast Pfu Polymerase, 0.2 μL bovine serum albumin, and 10 ng DNA extract. The PCR products were detected by electrophoresis on 2% agarose gel.

1. **Real-time quantitative PCR**

Real-time quantitative PCR, targeting the bacterial 16S gene, was conducted with the primer sets Eub338 forward primer (5’-ACT CCT ACG GGA GGC AGC AG-3’) and Eub806 reverse primer (5’-GGA CTA CHV GGG TWT CTA AT-3’). Each analysis consisted of a set of standards, Each analysis consisted of a set of standards, positive and negative controls and samples with 3 analytical replicates per sample on a 96-well plate. Melting curve analysis of PCR products was performed after each assay to determine the quality of PCR amplification (Fierer et al., 2005). Melting curve analyses were used to confirm the quality of PCR amplification quality (Zhang et al., 2017). Re-constructed plasmids were obtained by ligating the validated PCR products into the pMD-18T (2692bp, Takara Biotechnology Co., Ltd., Dalian, China) Easy vector for quantitative PCR, then the Blue-White Screening and plasmid DNA extraction were the performed. The plasmid OD_260_ values were measured using a UV spectrophotometer (NanoDrop 2000, Thermo Fisher Scientific, USA) and converted to gene copy number (copies /μl) to generate the qPCR standard curve. The amplification efficiency was 91.46%, and R^2^ value was 0.9914.

**Supplementary table 2 |** Supplementary PCR cycling conditions

| **Thermal Cycler** | **Times and Temperatures** | | | |
| --- | --- | --- | --- | --- |
|  | **Initial Steps** | **Each of 40 cycles** | | |
|  |  | Melt | Anneal | Extend |
| ABI 7300 (Applied Biosystems, USA) | Hold | **Cycle** | | |
|  | 3 min, 95℃ | 5 sec, 95℃ | 30 sec, 58℃ | 1 min, 72℃ |

1. **Illumina MiSeq sequencing**

Paired-end format of purified amplicons were mixed well and sequenced using an Illumina Miseq PE300 sequencing platform according to the standard protocols by Shanghai Majorbio Bio-pharm Technology Co., Ltd, Shanghai, China (Caporaso et al., 2011).Paired-end format of purified amplicons were mixed well and sequenced using an Illumina Miseq PE300 sequencing platform according to the standard protocols by Shanghai Majorbio Bio-pharm Technology Co., Ltd, Shanghai, China (Caporaso et al., 2011).


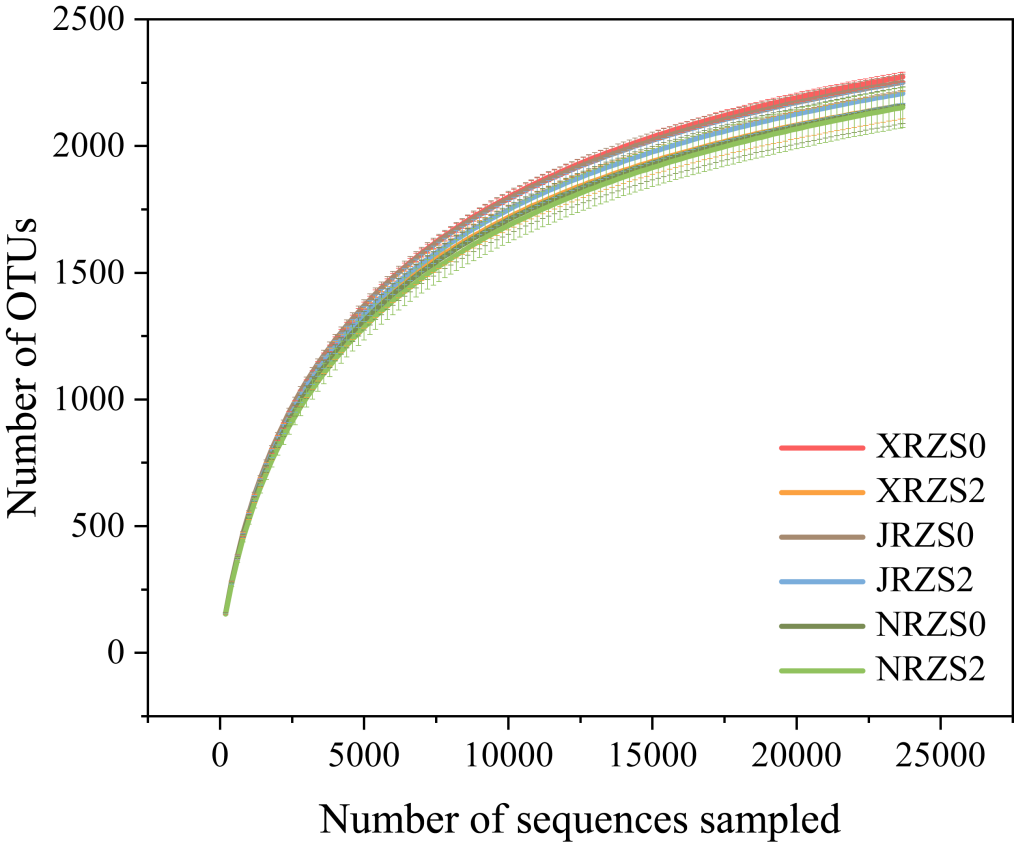


**Supplementary Figure 2 |** 16S rRNA sequencing rarefaction curve of rice rhizosphere soil.

**Part 3 Details of the co-occurrence network and topology roles of module nodes**

The abundance correlation between OTU species was analyzed in the samples, and it relied on the RANDOM MATRIX THEORY to calculate the adjacency matrix corresponding to the correlation matrix under different threshold conditions, which were adjusted until the eigenvalue distribution of the adjacency matrix approximated the Poisson distribution. Provided that the correlation coefficient exceeds the threshold, it is considered that there is a significant correlation between species so that it can determine the nodes and edges of the co-occurrence network.

The network characteristic parameters were then calculated, and the fast greedy method was adopted to implement the module clustering calculation, which calculated the node characteristic parameters (connection coefficients within modules, inter-module connection coefficients) and the module characteristic vectors (excluding calculating the modules with less than 5 nodes).

Microbial co-occurrence network topology characteristics allowed quantitative characterization and visualization of the bacterial community remodeling process (Fig. 3 and Table 3). The network connectivity distribution curves for all treatment groups conformed to the power-law model (0.79<R^2^<0.88), indicating the scale-free property of the network. In other words, fewer microorganisms in the MENs were more associated with other microorganisms, while most microorganisms were less associated with each other (Kong et al., 2019). The modularity index (M) for all treatment groups ranged from 0.781 to 0.911, which was significantly higher than the corresponding random network M values (0.476±0.004 to 0.636±0.004), indicating that all microbial co-occurrence networks were modular (Feng et al., 2017; Newman, 2006). Meanwhile, the average clustering coefficient (avgCC) and average path length (avgGD) of the empirical networks of all treatment groups were significantly higher than those of the random networks, and these characteristics were similar to other typical small-world networks (Watts and Strogatz, 1988), indicating that the obtained networks all had small-world characteristics.

To clarify the potential keystone genera based on topological roles in their networks, nodes were distinguished into Peripherals, Connectors, Module hubs, and Network hubs based on the connectivity of intra- and inter-modules in their co-occurrence networks (Guimerà and Amaral, 2005). The role of nodes in the network was measured in terms of inter-module connectivity (*P_i_*) and intra-module connectivity (*Z_i_*) (Fig. 4). (1) Network hubs: nodes where *P_i_*>0.62 and *Z_i_*>2.5; (2) Module hubs (i.e., nodes that highly connect nodes within modules): nodes where *P_i_*≤0.62 and *Z_i_*>2.5; (3) Connectors (i.e., nodes that highly connect some modules): nodes where *P_i_*>0.62 and *Z_i_*≤2.5; (4) Peripherals (nodes that have a small number of connections and are largely connected to nodes within the module): nodes where *P_i_*≤0.62 and *Z_i_*≤ 2.5.

**Part 4 Supplementary results for bacteria community characteristic analysis**


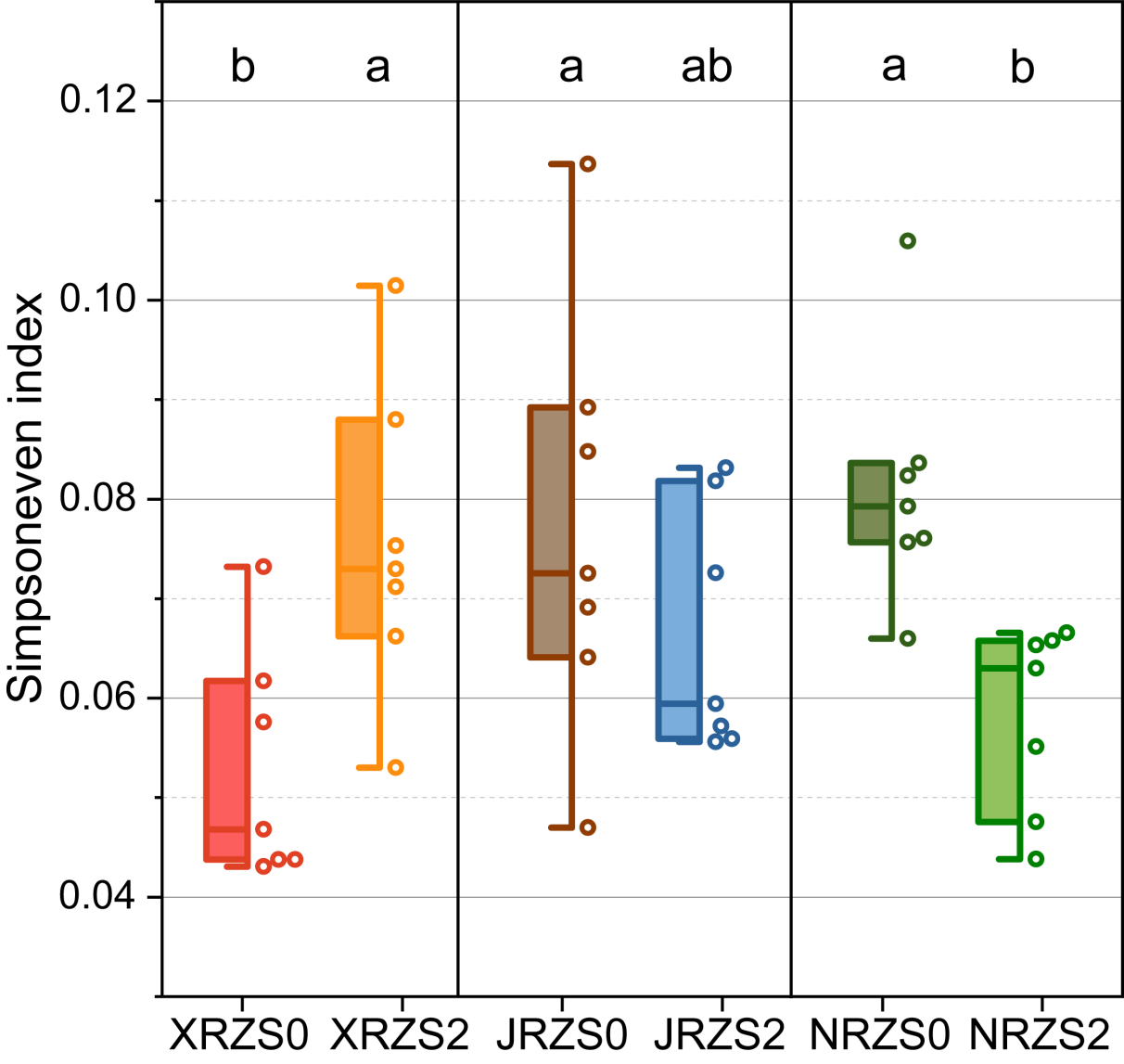


**Supplementary Figure 3 |** Simpson-evenness index of rhizosphere soil bacterial community diversity.


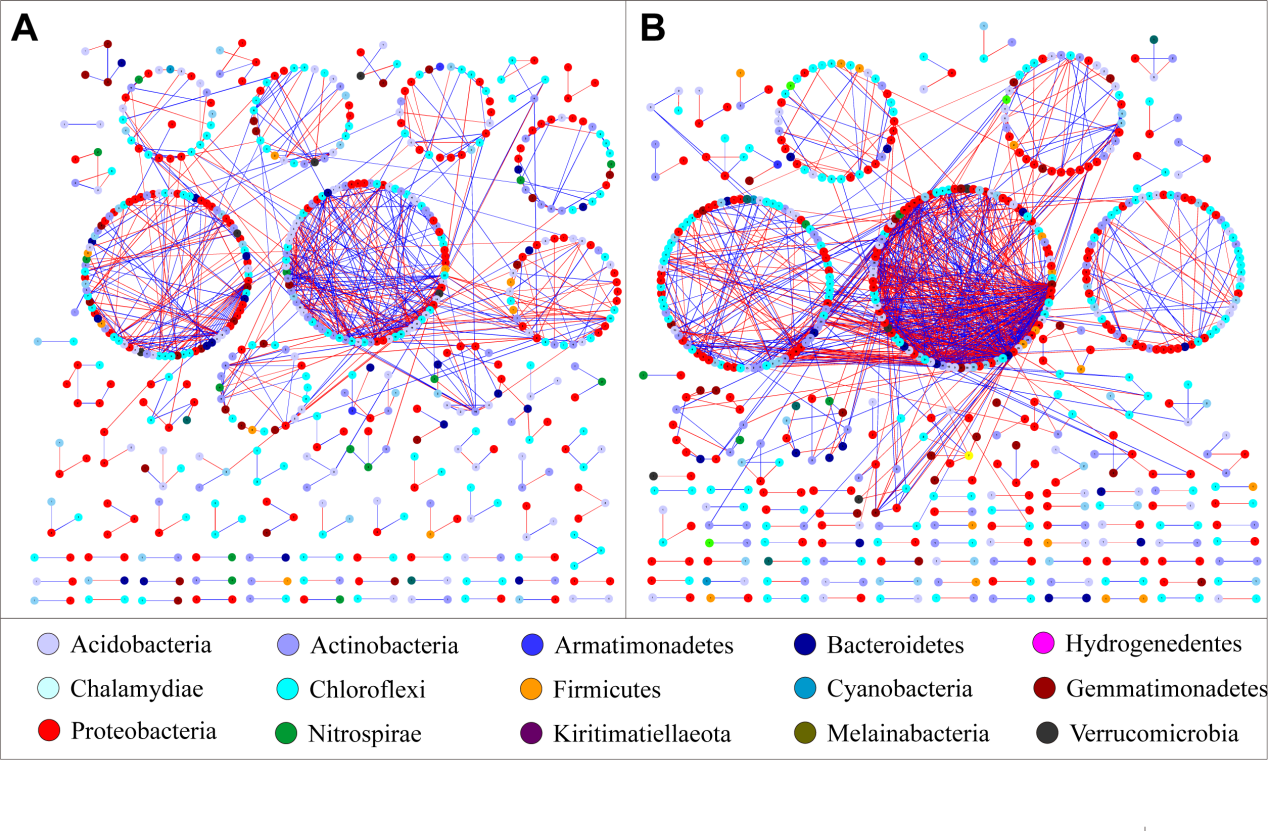


**Supplementary Figure 4 |** (A, B) Visual co-occurrence networks based on Modularity show the ZS0 and ZS2 treatments, respectively. The nodes' colors correspond to the taxonomic information at the phylum level; red links express positive interactions, and blue links stand for negative interaction.


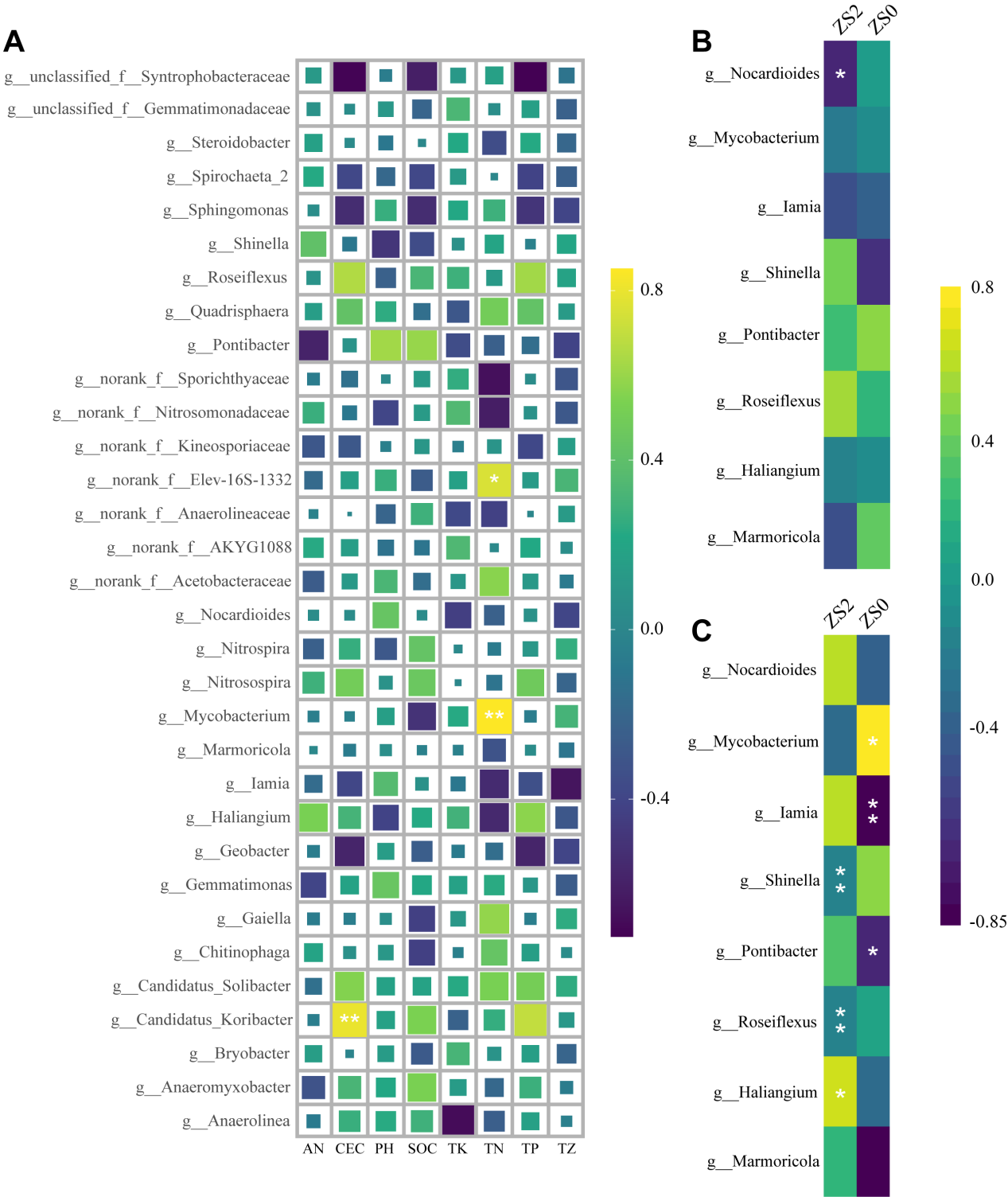


**Supplementary Figure 5 |** (A) Correlation (Pearson's coefficient) heat map between key rhizosphere soil bacteria (genus level) and soil chemical properties of rhizosphere soil; (B) Effect of soil zinc application on dominant key bacteria genus community structure of rice rhizosphere soil - zinc content in polished rice; (C) Effect of soil zinc application on dominant key bacteria genus community structure of rice rhizosphere - rice yields. * Denotes a significant difference (*p*<0.05, FDR adjust), and ** denotes a very significant difference (*p*<0.01, FDR adjust).


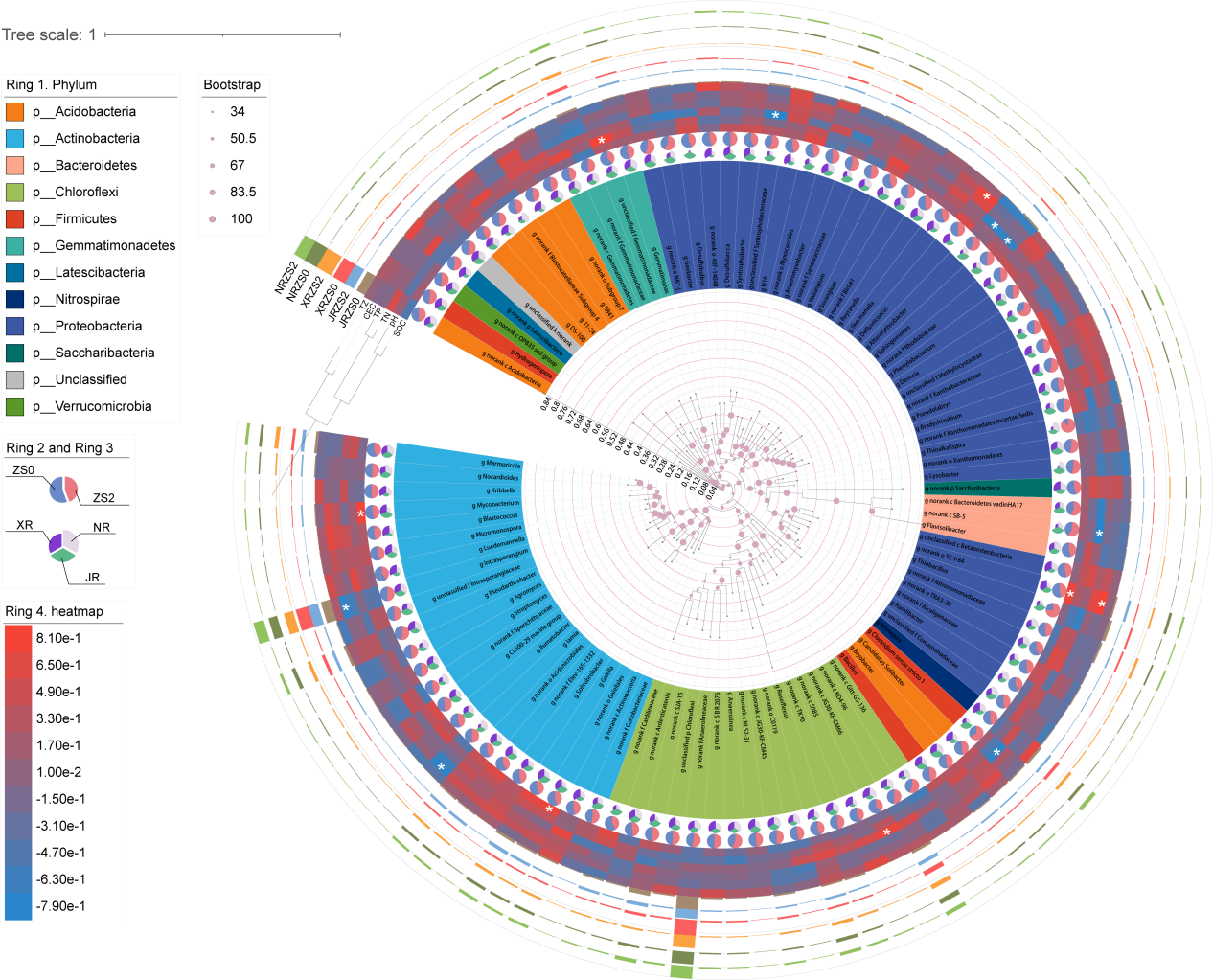


**Supplementary Figure 6 |** Phylogenetic distribution of the most abundant 100 bacterial taxon in the rice rhizosphere using the ML (Maximum Likelihood) method. The innermost ring 1 indicates bacterial classification based on phylum level. Rings 2 and 3 indicate the relative proportion of each bacterial taxa in different treatments and rice cultivars, respectively. The heatmap in ring 4 indicates correlation (Pearson's coefficient) heat map between bacteria and soil physicochemical properties. Absolute abundance of bacteria in each treatments are shown in ring 5 as barplots. The pink dots indicate bootstrap value. * Denotes a significant difference (*p*<0.05, FDR adjust).

**Supplementary table 3 |** Key species identified by *Z_i_*≥2.5 or *P_i_*≥0.62

| **Varieties** | **OTU** | **Phylum** | **Genus** | ***P_i_*** | ***Z_i_*** |
| --- | --- | --- | --- | --- | --- |
| XRZS0 | OTU7881 | *Actinobacteria* | g__Mycobacterium | 0 | 2.597 |
|  | OTU3437 | *Actinobacteria* | g__CL500-29_marine_group | 0 | 2.649 |
|  | OTU8281 | *Actinobacteria* | g__Iamia | 0 | 2.597 |
|  | OTU7630 | *Chloroflexi* | g__norank_f__Anaerolineaceae | 0 | 2.657 |
|  | OTU3925 | *Chloroflexi* | g__Anaerolinea | 0 | 3.153 |
|  | OTU7468 | *Bacteroidetes* | g__Pontibacter | 0.64 | -0.947 |
|  | OTU8055 | *Bacteroidetes* | g__Pontibacter | 0.625 | -0.224 |
|  | OTU8921 | *Ignavibacteriae* | g__norank_o__Ignavibacteriales | 0.666 | -1.261 |
|  | OTU5738 | *Nitrospirae* | g__Nitrospira | 0 | 2.974 |
|  | OTU6723 | *Proteobacteria* | g__unclassified_f__Syntrophobacteraceae | 0 | 2.582 |
|  | OTU2753 | *Chlorobi* | g__norank_f__SJA-28 | 0 | 2.639 |
| XRZS2 | OTU9165 | *Actinobacteria* | g__norank_f__Kineosporiaceae | 0.653 | -0.98 |
|  | OTU6415 | *Actinobacteria* | g__norank_f__0319-6M6 | 0.64 | -0.959 |
|  | OTU8136 | *Actinobacteria* | g__norank_f__Sporichthyaceae | 0 | 2.637 |
|  | OTU3631 | *Actinobacteria* | g__Nocardioides | 0 | 2.637 |
|  | OTU2247 | *Acidobacteria* | g__Candidatus_Koribacter | 0 | 2.637 |
|  | OTU5568 | *Acidobacteria* | g__norank_c__Acidobacteria | 0 | 2.637 |
|  | OTU1549 | *Gemmatimonadetes* | g__unclassified_f__Gemmatimonadaceae | 0.64 | -1.224 |
|  | OTU3714 | *Gemmatimonadetes* | g__norank_c__Gemmatimonadetes | 0 | 2.571 |
|  | OTU7961 | *Chloroflexi* | g__norank_o__C0119 | 0.625 | -1.363 |
|  | OTU8921 | *Ignavibacteriae* | g__norank_o__Ignavibacteriales | 0.653 | -0.959 |
|  | OTU8072 | *Nitrospirae* | g__Nitrospira | 0 | 2.723 |
| JRZS0 | OTU6572 | *Proteobacteria* | g__norank_f__Nitrosomonadaceae | 0.666 | -1.279 |
|  | OTU519 | *Proteobacteria* | g__norank_f__Nitrosomonadaceae | 0 | 2.545 |
|  | OTU549 | *Proteobacteria* | g__norank_o__NB1-j | 0 | 2.777 |
|  | OTU7195 | *Acidobacteria* | g__norank_c__Acidobacteria | 0 | 2.545 |
|  | OTU7272 | *Acidobacteria* | g__DS-100 | 0 | 2.57 |
|  | OTU1922 | *Actinobacteria* | g__norank_f__Elev-16S-1332 | 0 | 2.88 |
|  | OTU9669 | *Chloroflexi* | g__norank_f__Anaerolineaceae | 0 | 2.611 |
|  | OTU1921 | *Bacteroidetes* | g__Chitinophaga | 0 | 2.611 |
| JRZS2 | OTU4078 | *Acidobacteria* | g__norank_c__Acidobacteria | 0 | 2.937 |
|  | OTU3715 | *Acidobacteria* | g__norank_c__Acidobacteria | 0 | 2.516 |
|  | OTU2630 | *Proteobacteria* | g__Steroidobacter | 0 | 2.89 |
|  | OTU1054 | *Proteobacteria* | g__Shinella | 0 | 2.561 |
|  | OTU8081 | *Chloroflexi* | g__unclassified_p__Chloroflexi | 0 | 2.585 |
|  | OTU3407 | *Chloroflexi* | g__norank_c__SBR2076 | 0 | 2.889 |
|  | OTU8228 | *Nitrospinae* | g__norank_c__Belgica2005-10-ZG-3 | 0 | 2.89 |
| NRZS0 | OTU8023 | *Chloroflexi* | g__unclassified_p__Chloroflexi | 0 | 2.743 |
|  | OTU9085 | *Spirochaetae* | g__Spirochaeta_2 | 0 | 2.813 |
|  | OTU7122 | *Proteobacteria* | g__Nitrosospira | 0 | 2.87 |
| NRZS2 | OTU2010 | *Proteobacteria* | g__norank_f__Acetobacteraceae | 0 | 2.608 |
|  | OTU7860 | *Proteobacteria* | g__norank_f__AKYG1088 | 0.244 | 2.529 |
|  | OTU8636 | *Proteobacteria* | g__Geobacter | 0 | 3.175 |
|  | OTU8613 | *Acidobacteria* | g__norank_c__Acidobacteria | 0.625 | -0.673 |
|  | OTU4656 | *Actinobacteria* | g__Quadrisphaera | 0 | 2.658 |
|  | OTU5583 | *Chloroflexi* | g__norank_c__S085 | 0.625 | -0.936 |
|  | OTU7111 | *RBG-1__Zixibacteria_* | g__norank_p__RBG-1__Zixibacteria_ | 0.625 | -0.59 |
|  | OTU3733 | *Chloroflexi* | g__norank_o__JG30-KF-CM45 | 0.64 | -1.387 |

**Reference**

Feng K, Zhang ZJ, Cai WW, Liu WZ, Xu MY, Yin HQ, Wang AJ, He ZL, Deng Y (2017). Biodiversity and species competition regulate the resilience of microbial biofilm community. ***MOLECULAR ECOLOGY***, 26(21): 6170-6182. https://doi.org/10.1111/mec.14356.

Fierer N, Jackson JA, Vilgalys R, Jackson RB (2005). Assessment of soil microbial community structure by use of taxon-specific quantitative PCR assays. ***APPLIED AND ENVIRONMENTAL MICROBIOLOGY***, 71(7): 4117-4120. https://doi.org/10.1128/AEM.71.7.4117-4120.2005.

Guimera R, Amaral LAN (2005). Cartography of complex networks: modules and universal roles. ***JOURNAL OF STATISTICAL MECHANICS-THEORY AND EXPERIMENT***, P02001. https://doi.org/10.1088/1742-5468/2005/02/P02001.

Kong X, Jin DC, Wang XX, Zhang FS (2019). Dibutyl phthalate contamination remolded the fungal community in agro-environmental system. ***CHEMOSPHERE***, 215: 189-198. https://doi.org/10.1016/j.chemosphere.2018.10.020.

Newman MEJ (2006). Modularity and community structure in networks. ***PROCEEDINGS OF THE NATIONAL ACADEMY OF SCIENCES OF THE UNITED STATES OF AMERICA***, 103(23): 8577-8582. https://doi.org/10.1073/pnas.0601602103.

Watts DJ, Strogatz SH (1998). Collective dynamics of 'small-world' networks. ***NATURE***, 393(6684): 440-442. https://doi.org/10.1038/30918.

Zhang XX, Zhang RJ, Gao JS, Wang XC, Fan FL, Ma XT, Yin HQ, Zhang CW, Feng K, Deng Y (2017). Thirty-one years of rice-rice-green manure rotations shape the rhizosphere microbial community and enrich beneficial bacteria. ***SOIL BIOLOGY & BIOCHEMISTRY***, 104: 208-217. https://doi.org/10.1016/j.soilbio.2016.10.023.
